# A Model of Filamentous Cyanobacteria Leading to Reticulate Pattern Formation 

**DOI:** 10.3390/life4030433

**Published:** 2014-09-02

**Authors:** Carlos Tamulonis, Jaap Kaandorp

**Affiliations:** Section for Computational Science, Faculty of Science, University of Amsterdam, Science Park 402, 1098 XH Amsterdam, The Netherlands; E-Mail: ctamulon@gmail.com

**Keywords:** filamentous cyanobacteria, computational modeling, stromatolites

## Abstract

The filamentous cyanobacterium, *Pseudanabaena*, has been shown to produce reticulate patterns that are thought to be the result of its gliding motility. Similar fossilized structures found in the geological record constitute some of the earliest signs of life on Earth. It is difficult to tie these fossils, which are billions of years old, directly to the specific microorganisms that built them. Identifying the physicochemical conditions and microorganism properties that lead microbial mats to form macroscopic structures can lead to a better understanding of the conditions on Earth at the dawn of life. In this article, a cell-based model is used to simulate the formation of reticulate patterns in cultures of *Pseudanabaena*. A minimal system of long and flexible trichomes capable of gliding motility is shown to be sufficient to produce stable patterns consisting of a network of streams. Varying model parameters indicate that systems with little to no cohesion, high trichome density and persistent movement are conducive to reticulate pattern formation, in conformance with experimental observations.

## 1. Introduction

The Myxobacteria fruiting body and the slug formed by starving *Dictyostelium discoideum* amoebae are two widely known examples of morphogenesis (see [1,2] for reviews). These fascinating systems, which seem to teeter on the boundary between unicellular and multicellular modes of life, are initially composed of independent unicells that coordinate their actions using cell signaling (c-signaling in Myxobacteria and cAMP in *D. discoideum*). Despite their apparent simplicity, these systems display rich self-organizing behavior culminating in a spatially differentiated multi-cellular body. In this article, we explore a little-known system in the field of morphogenesis, which is simpler and much older than either amoebae or Myxobacteria: gliding filamentous cyanobacteria.

Cyanobacteria form a large and diverse phylum of prokaryotes that is chiefly defined by the ability to perform oxygen-producing photosynthesis [3]. They play an extremely important role in the global eco-system by fixating carbon and nitrogen, providing a large part of the planet’s oxygen and the base of aquatic food webs. They can be planktonic or fixed in biofilms and benthic algal mats. They are also found in extreme environments, such as deserts, the polar regions and hot springs.

**Figure 1:**
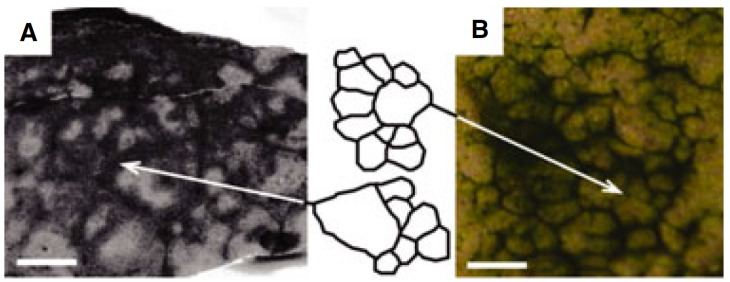
Comparison of in vitro reticulate patterns with ancient fenestrate microbialites from the 2.52-Ga Gamohaan Formation, South Africa. (**A**) Horizontal cross-section of the basal layer of a fenestrate microbialite. The arrow indicates the left edge of the tracing of the reticulate structure. Scale bar = 1 cm; (**B**) Experimental mat showing peaks and ridges at the same scale. The arrow indicates the right edge of the tracing. Note the similarities in structure between the ancient and experimental structures. Scale bar = 1 cm. Adapted from [7] with permission from John Wiley & Sons Ltd.

Non-branching filamentous cyanobacteria are linear arrays of individual cells that form long and thin flexible trichomes (if the trichome has a sheath, it is referred to as a filament). Motile forms are capable of gliding, which occurs when the trichomes are attached to a (semi-) solid object [4]. Walter *et al.* [5] first demonstrated that filamentous cyanobacteria could produce macroscopic structures when cultured and suggested that ancient cyanobacteria may have built similar structures found in the fossil record. More recently, Petroff *et al.* [6] linked the morphology of these cone-shaped structures to the metabolism of the cyanobacteria that forms them. In another study, Shepard *et al.* [7] documented pattern formation in cultures of the filamentous cyanobacterium, *Pseudanabaena*. They showed that in cultures with sufficient trichome density, the trichomes would organize within a matter of hours into a pattern of connected ridges defining a distinct polygonal reticulum (Figure 1). They postulated that the patterns resulted solely from the random motility of the trichomes and that no form of signaling was required. They further speculated that reticulate patterns found in the fossil record may be due to a similar process.

These patterns, both fossil and extant, are important, since they are associated with stromatolites—laminated sedimentary rock formed by the trapping and binding of sediment by filamentous cyanobacteria—which constitute the oldest evidence of life on Earth, the oldest dating to at least 3.4 Ga (billion years ago [8]). In hot-springs in Yellowstone National Park and New Zealand and in Antarctic lakes, reticulate patterns are found in the form of ridges connected to small cones or tepee-like structures [5,9,10]. Fossilized forms of these extant patterns have been found in some of the the oldest stromatolitic outcrops known ([8], Figure 1), and the Yellowstone cones in particular have been proposed to be modern day analogues of much larger ancient stromatolites, called *Conophyton* [5]. Intriguingly, although filamentous cyanobacteria are ubiquitous in microbial mats found all over the planet [11], the formation of conical structures and reticulate mats appears to be uncommon. Identifying the specific physicochemical conditions and microorganism properties that lead microbial mats to form macroscopic structures can lead to a better understanding of the conditions on Earth at the dawn of life [6,12,13].

In this article, we use a three-dimensional cell-based model to simulate the formation of reticulate patterns in cultures of filamentous gliding cyanobacteria. Our initial objective is limited in scope to testing and expanding on Shepard *et al.*’s hypotheses and studying the effect of varying the trichome parameters on pattern formation. The long-term goal, however, is to understand how the characteristics of stromatolite-building microorganisms and physicochemical conditions combine to form stromatolites, both ancient and contemporary.

We used an extension of a prior two-dimensional model of gliding filamentous cyanobacteria for our study [14]. Three-dimensional cell-based models of bacteria have also been used for simulating Myxobacteria [15,16], including fruiting body formation [17], *E. coli* [18] and biofilms composed of coccoidal cells [19]. Systems of “elongated self-propelled rods” have also generated interest in physics for their self-alignment and swarming behavior, leading to a theoretical model of these systems analogous to the theory of liquid crystals [20,21]. Many computational models of *D. discoideium* and *M. xanthus*’s fruiting body formation have been published, of which [22,23] are notable examples, respectively.

## 2. Methods

### 2.1. Biological Background

Cyanobacteria often form colonies with a multitude of forms, from square blocks to extremely long filaments that may be branching or linear, straight or spiral [24]. The cells within these colonies may be disk-shaped, cylindrical or even spherical, and cells may differentiate within a colony to perform a specific function, such as heterocysts specialized in nitrogen fixation.

Here, we focus on non-branching filamentous forms of cyanobacteria, which have been found to drive pattern formation [5,7,9,14,25]. Pattern formation in these species is driven by gliding motility—a form of cell movement that occurs in the presence of a substrate and that does not rely on any obvious external organ or change in cell shape [24,26]. Gliding in filamentous cyanobacteria appears to be enabled by a “slime jet” mechanism, similar to that found in Myxobacteria, in which the cells extrude a gel through a set of pores. The gel expands quickly as it hydrates, providing a propulsion force [4,27]. Just as in the Myxobacteria, each cell has two sets of pores, one at each end. However, since filamentous cyanobacteria are composed of an array of squat cells, and not a single elongated cell, as the Myxobacteria, there are pores located all along the trichome at the cell junctions producing a tangential force along the length of the trichome. The cells appear to coordinate their gliding direction by an electrical potential that establishes polarity in the trichomes and, thus, establishes a “head” and a “tail” [28]. Based on the polarity, only one set of pores in each cell is active, so the cells all push in the same direction. Trichomes usually reverse their polarity randomly with an average period on the order of minutes to hours [25,29]. However, depending on the incident light, cyanobacteria can adjust their reversal period, so that they move into more favorable lighting conditions, a phenomenon called photomovement [25,30,31]. Being photoautotrophs, cyanobacteria depend on light for survival, yet intense radiation can inhibit photosynthesis or even kill the cells. Using a combination of photokinesis (gliding speed is a function of light intensity), phototaxis (gliding direction is a function of the incident light direction) and photophobia (gliding direction is a function of the temporal and spatial gradient of the light field), cyanobacteria can position themselves with great accuracy in their environment [14,25,32,33,34,35].

Photomovement was thought to be essential for pattern formation in cyanobacterial mats and cultures [5]. However, the formation of reticulate patterns in cultures of *Pseudanabaena* is likely to be a result of pure unbiased random gliding movement [7], since reticulates form in uniform lighting conditions (although self-shading may still have some effect). Therefore, we chose to ignore the effects of photomovement and assume that the trichomes reverse polarity with a constant frequency.

### 2.2. Model Geometry

We used a cell-based model in which trichomes are represented individually. Every trichome in the model has length Λ and diameter Θ and is represented as a discrete chain of *N* edges of equal length *l* = Λ*/N* for a total of *N* + 1 vertices. The dynamics of the system are inertialess and are given by the set of first order ordinary differential equations:

(1) $$\frac{dx_{i}}{dt}=\frac{1}{\zeta_{i}}\left(F_{i}^{∥}+\frac{1}{b}F_{i}^{⊥}\right),$$

where **x***_i_* is the position of the vertex relative to the domain origin, *ζ_i_* is the drag experienced by the vertex and $F_{i}^{∥}$ and $F_{i}^{⊥}$ are the tangential and perpendicular components, respectively, of the total force acting on the vertex given by:

(2) $$F_{i}^{∥}=\left(F_{i}\cdot {\hat{t}}_{i}\right){\hat{t}}_{i},\;F_{i}^{⊥}=F_{i}-F_{i}^{∥},$$

where ${\hat{t}}_{i}$ is a unitary vector tangent to the trichome at vertex *i*. For long and thin trichomes, *b* *≃* 2 [36]. Note that for simplicity, we use a single index *i* on variables to denote the *i*-th of *N* + 1 vertices in a trichome centerline and omit an extra index *f* to denote the *f* -th trichome in the system, although the extra index is implied.

The total force on each vertex is the sum of forces arising from elasticity, gliding and contact:

(3) $$F_{i}=F^{e}\left(x_{i-2},x_{i-1},x_{i},x_{i+1},x_{i+2}\right)+F^{g}\left(x_{i-1},x_{i},x_{i+1}\right)+{\sum_{k\in \text{nhd}(i)}F}^{c}\left(x_{i},x_{i+1},x_{k},x_{k+1}\right).$$

### 2.3. Elasticity

To simulate trichome elasticity, we use the framework proposed by Bergou *et al.* [37] for modeling discrete elastic rods. Their framework allows the trichomes to be represented by their centerlines using Cartesian coordinates, unlike other models, which are based on curvature [38]. Using Cartesian coordinates facilitates coupling the elasticity equations with collisions and other processes.

Bergou *et al.*’s model assumes that the rod is inextensible, but allows for bending and torsion. Here, we ignore torsion and enforce the inextensibility constraint by using the Linear Constraint Solver (LINCS) algorithm commonly used in molecular dynamics simulations [39]. For a naturally straight and inextensible rod, the force on each vertex is given by the sum of up to three contributions [37]:

(4) $$F^{e}=-\sum_{j=i-1}^{i+1}\frac{2\alpha }{l}{\left(\nabla_{i}{\left(\kappa b\right)}_{j}\right)}^{T}{\left(\kappa b\right)}_{j},$$

where *α* is the bending modulus of the trichome, *l* is the length of the edges that compose the model trichome and (*κ***b**)*_j_* is called the discrete curvature binormal and is a vector perpendicular to the osculating plane between two consecutive edges whose magnitude is equal to the discrete curvature of the trichome at vertex *j*:

(5) $${\left(\kappa b\right)}_{i}=\frac{2e_{i-1}\times e_{i}}{∥e_{i-1}∥∥e_{i}∥+e_{i-1}\cdot e_{i}}.$$

The gradient of the curvature binormal is given by:

(6) $$\nabla_{i-1}{\left(\kappa b\right)}_{i}=\frac{2\left[e_{i}\right]+\left(\kappa b_{i}\right){\left(e_{i}\right)}^{T}}{|e_{i-1}||e_{i}|+e_{i-1}\cdot e_{i}},$$

(7) $$\nabla_{i+1}{\left(\kappa b\right)}_{i}=\frac{2\left[e_{i-1}\right]+\left(\kappa b_{i}\right){\left(e_{i-1}\right)}^{T}}{|e_{i-1}||e_{i}|+e_{i-1}\cdot e_{i}},$$

(8) $$\nabla_{i}{\left(\kappa b\right)}_{i}=-\left(\nabla_{i-1}+\nabla_{i+1}\right){\left(\kappa b\right)}_{i},$$

where **e***_i_* = **x***_i_*_+1_*−* **x***_i_*, [**e**] is a 3 *×* 3 matrix such that [**e**] *·* **x** = **e** *×* **x**.

### 2.4. Gliding

Gliding in filamentous cyanobacteria is thought to be powered by a “slime-jet” mechanism. Thrust is generated by the extrusion of a dehydrated gel through pores organized in rings at either end of every cell in the trichome [4]. The expansion of the gel upon hydration generates a tangential force that propels the trichome forward. We make no assumption about the specific mechanism of gliding; we simply assume that a tangential force (**F**^g^) is applied along the length of the trichome with a magnitude sufficient to propel the trichome at the target speed (*v*_0_). Gliding requires contact with some (semi-) solid substrate in order to provide a reaction force to the gliding mechanism. We assume that the trichomes are immersed in a highly viscous medium that allows them to glide freely in all directions. This medium could consist of the EPSthe trichomes produce copiously when gliding [4]. Gliding cyanobacteria also frequently reverse their gliding direction [25]. We assume that each trichome has a polarization *P* *∈* {*−*1*,* 1} that determines the direction in which it is gliding. *P* is a stochastic process drawn at each simulation step for each trichome and is such that the trichomes reverse direction with an average frequency *ω*. This is simulated by generating a pseudo-random number *x* following a uniform distribution *X* *∼* *U* (0*,* 1) and reversing the gliding direction if *x < ω*∆*t*. The gliding force is given by:

(9) $$F_{i}^{g}=\zeta_{i}v_{0}P{\hat{t}}_{i},$$

where ${\hat{t}}_{i}$ is a unitary vector tangent to the filament at the vertex **x***_i_*:

(10) $${\hat{t}}_{i}=\{\begin{array}{c} \frac{\left(x_{i+1}-x_{i}\right)}{∥x_{i+1}-x_{i}∥},\;i=0\\ \frac{\left(x_{i+1}-x_{i-1}\right)}{∥x_{i+1}-x_{i-1}∥},\;0<i<N\\ \frac{\left(x_{i-1}-x_{i}\right)}{∥x_{i-1}-x_{i}∥},\;i=N \end{array}$$

### 2.5. Contact Interaction

**Figure 2:**
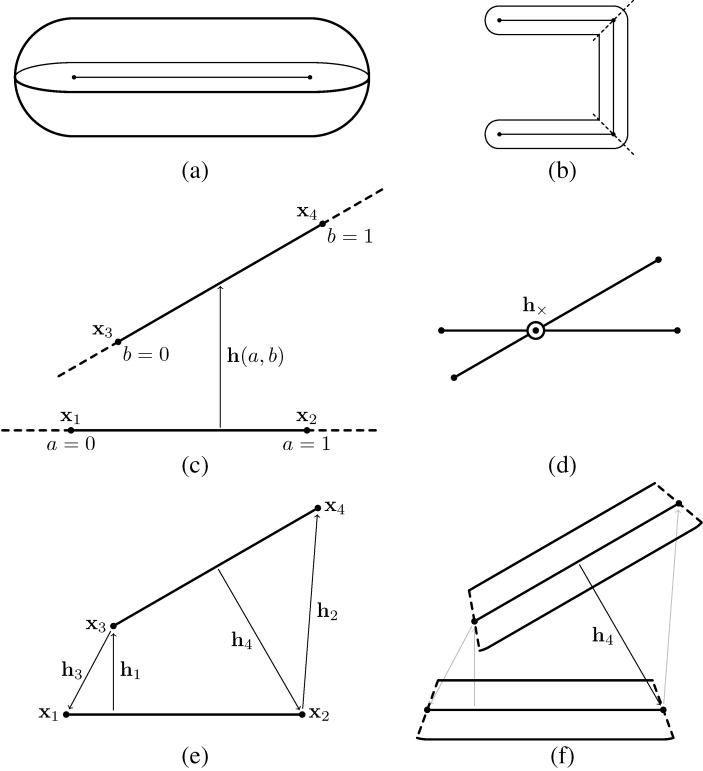
Modeling contact interactions between trichomes. (**a**) Individual segments are capsule-shaped and are defined parametrically, based on an edge; (**b**) trichomes consist of a sequence of linked segments, such that the underlying edges form a linear polyline. The domain of each segment is determined by “cutting” the segment at each of its vertices perpendicular to the local tangent; (**c**) Contact interactions are defined between pairs of edges. A simple linear parameterization h(a; b) is used for the interaction vector between the edges; (**d**) For any two non-colinear edges, there is a plane that is parallel to both. If the edges cross each other when projected onto this plane, then the interaction will be along the vector connecting the two edges at the interaction point; (**e**) For edges that do not cross, the four possible interactions between any vertex and the opposing edge are considered; (**f**) Only interactions that are “contained” within the domain of both segments are applied (only h4 in this example).

Trichomes are modeled as three-dimensional articulated filaments, each of which is represented by a chain of edges that forms its centerline. The edges are of equal length, with each edge representing a segment of a trichome. The domain of each segment is based on a straight cylinder with a semi-spherical cap at both ends, forming a capsule shape (Figure 2a). For the sake of modeling contact interactions, the trichomes are assumed to flex only at the vertices. The domain of each segment is determined by “cutting” the trichome at each vertex in the direction perpendicular to the tangent, except for the first and last vertices, which are assumed to be capped with semi-spheres (Figure 2b). Thus, the domain of a given trichome segment is a function of its two vertices and and tangent vectors at those vertices. For a completely straight trichome, the domain of every segment is effectively a cylinder, except of the ends, which are capped with semi-spheres. Otherwise, when two segments form an angle, the domain of either segment will be rounded on the outside of the angle, but sharp on the inside.

Let 〈**x**_1_*,***x**_2_〉 and 〈**x**_3_*,***x**_4_〉 be any two edges in the model (Figure 2c). We parameterize points on the first and second edges by the scalars *a* and *b*, respectively, such that any displacement vector between the edges can be given by:

**h**(*a, b*) = (*b***x**_4_+ (1 *−b*)**x**_3_) *−* (*a***x**_2_+ (1 *−a*)**x**_1_)*,* 0 *≤a, b≤* 1 (11)

Segments interact via steric and cohesion forces applied at discrete contact points 〈*a, b*〉 where the edges come into close range (Figure 2c). The forces resulting from an interaction between a pair of points 〈*a, b*〉 are given by a Lennard–Jones type interaction, following [15,40]. The Lennard–Jones interaction captures the strong repulsion for colliding segments and attraction for edges within a close vicinity:

(12) $$F^{c}=12\epsilon \left\{{\left(\frac{\Theta }{h}\right)}^{13}-{\left(\frac{\Theta }{h}\right)}^{7}\right\}\hat{h},$$

where *h* = *‖***h**(*a, b*)*‖*, $\hat{h}$ = **h**(*a, b*)*/h*, Θ is the trichome diameter and *ϵ* is the cohesion strength. The strong repulsion forces are meant to mimic the reaction forces of two rigid bodies colliding, whereas the attractive forces are meant to simulate the cohesion between trichomes due to the sticky slime they secret while gliding. The attractive force has a short range and is practically zero at *h* = 2ϴ. On the other hand, the repulsive force is unbounded as *h* *→* 0, and so, to reduce numerical stiffness, the intensity of the repulsive force was capped to a maximum value of *R*.

Since we represent each edge by its two vertices, any interaction force between edges must be translated into “equivalent” forces on the vertices by linear interpolation, assuming the segments are rigid. In terms of our parameterization, a contact force **F**^c^ between points 〈*a, b*〉 becomes:

(13) $$\begin{array}{c} F_{1}^{c}=(1-a)F^{c},\;F_{2}^{c}=aF^{c},\\ F_{3}^{c}=-(1-b)F^{c},\;F_{4}^{c}=-bF^{c}. \end{array}$$

The sum of the interaction’s opposing forces and torques is zero, thereby ensuring that Newton’s third law is respected.

To determine the direction of the interaction forces between two edges, first we calculate *a_×_,b_×_* corresponding to the closest points between the lines the edges lie on (Figure 2d):

(14) $$\begin{array}{c} a_{\times }=-\frac{r_{34}\cdot r_{34}\times r_{13}\cdot r_{12}-r_{12}\cdot r_{34}\times r_{13}\cdot r_{34}}{r_{12}\cdot r_{34}\times r_{12}\cdot r_{34}-r_{12}\cdot r_{12}\times r_{34}\cdot r_{34}}\\ b_{\times }=-\frac{r_{12}\cdot r_{34}\times r_{13}\cdot r_{12}-r_{12}\cdot r_{12}\times r_{13}\cdot r_{34}}{r_{12}\cdot r_{34}\times r_{12}\cdot r_{34}-r_{12}\cdot r_{12}\times r_{34}\cdot r_{34}} \end{array}$$

where **r***_ij_* = **x***_j_−* **x***_i_*. If 0 *< a_×_,b_×_* *<* 1, then the edges cross each other, and the interaction vector between the crossing points **h***_×_* = **h**(*a_×_,b_×_*) is the sole interaction considered.

If the edges do not cross, we consider four possible interaction vectors between an edge and an opposing vertex (Figure 2e):

(15) $$\begin{array}{c} h_{1}=h(g(x_{1},x_{2},x_{3}),0),\\ h_{2}=h(g(x_{1},x_{2},x_{4}),1),\\ h_{3}=h(0,g(x_{3},x_{4},x_{1})),\\ h_{4}=h(1,g(x_{3},x_{4},x_{2})), \end{array}$$

where *g*(**x***_i_,* **x**_j_*,* **x***_k_*) gives the closest point on the edge 〈**x***_i_,***x***_j_*〉 to the point **x***_k_*:

(16) $$g(x_{i}\text{,}x_{j}\text{,}x_{k})=\text{clamp}\left(\frac{r_{ik}\cdot r_{ij}}{r_{ij}\cdot r_{ij}}\right),$$

where:

(17) $$\text{clamp}(x)=\left\{ \begin{array}{l} 1,\;x>1\\ 0,\;x<0\\ x,\text{ otherwise} \end{array}\right.$$

Finally, only the interactions that are consistent with the segment domains are considered. The domain conditions for each possible interaction vector are (Figure 2f):

(18) $$\begin{array}{l} h_{\text{1}}\text{: }r_{\text{13}}\cdot t_{\text{1}}\geq \text{0 and }r_{\text{23}}\cdot t_{\text{2}}<0\text{ and }(h_{\text{1}}\cdot t_{\text{3}}\leq \text{0 or }x_{\text{3}}\text{ is an end vertex),}\\ h_{2}\text{: }r_{\text{14}}\cdot t_{\text{1}}\geq 0\text{ and }r_{\text{24}}\cdot t_{\text{2}}<0\text{ and }(h_{2}\cdot t_{4}>0,\text{ or }x_{4}\text{ is an end vertex),}\\ h_{3}\text{: }r_{\text{31}}\cdot t_{3}\geq 0\text{ and }r_{41}\cdot t_{4}<0\text{ and }(h_{3}\cdot t_{1}\leq 0\text{ or }x_{1}\text{ is an end vertex),}\\ h_{4}\text{: }r_{32}\cdot t_{3}\geq 0\text{ and }r_{42}\cdot t_{4}<0\text{ and }(h_{4}\cdot t_{2}>\text{0 or }x_{2}\text{ is an end vertex),} \end{array}$$

where the **t***_i_* are the tangent vectors, as defined in Equation (10). Of the four pairs, in practice, at most two will result in a non-null force.

### 2.6. Boundary and Initial Conditions

The trichomes are set in a cuboidal domain of dimensions *W* *×* *H* *×* *D*. The dimensions are chosen such that the domain is shallow, *i.e.*, *H* «*W* = *D*. The boundaries perpendicular to the **e***_x_,***e***_y_* directions (the sides) are periodic, but the boundaries perpendicular to the **e***z* direction (top and bottom) are “hard”. Trichomes pressed against the top or bottom planes are repulsed by a reaction force on their vertices:

(19) $$F^{b}(z)\left\{ \begin{array}{l} c\text{(}H-\Theta \text{/2}-{\text{z)e}}_{z},z>H-\Theta /2\\ c\text{(}\Theta \text{/2}-{\text{z)e}}_{z}\text{, }z<\Theta /2\\ 0\text{, otherwise} \end{array}\right.$$

where *z* is the height coordinate of the vertex and *c* is a constant sufficiently large to make the boundaries stiff. This force has not been included in Equation (3) for the sake of succinctness.

The trichomes are initially set parallel to the *xy* plane with a random orientation and a random height. The model is then run with only the repulsive steric forces turned on, until the system reaches an equilibrium in which there are no overlaps between trichomes.

### 2.7. Nondimensionalization

In this section, we nondimensionalize the model equations to determine the core set of model parameters. First, we normalize the position and time variables using a characteristic length *L* and time *T* of our choosing:

(20) X=x/L, τ=t/T,

then we write the equations in non-dimensional form:

(21) $$\frac{L}{T}\frac{dX}{d\tau }=\frac{1}{\zeta }\left(\frac{\alpha }{L^{2}}F^{b}+\zeta v_{0}F^{g}+\epsilon F^{c}\right),$$

where we have separated the parameters from the forces, which are now only functions of the positions. The 1*/L*^2^ factor on the bending force is the result of substituting **X** for **x**. Rearranging, we have:

(22) $$\frac{dX}{d\tau }=\frac{T\alpha }{L^{3}\zeta }F^{b}+\frac{Tv_{0}}{L}F^{g}+\frac{T\epsilon }{L\zeta }F^{c}.$$

By defining the characteristic velocity *V* = *L/T* and substituting, we have:

(23) $$\frac{dX}{d\tau }=\frac{\alpha }{VL^{2}\zeta }F^{b}+\frac{v_{0}}{V}F^{g}+\frac{\epsilon }{V\zeta }F^{c}.$$

We choose the characteristic velocity and length of the system to be:

(24) $$V=v_{0},\;L=\sqrt{\frac{\alpha }{v_{0}\zeta }}.$$

so that the first and second coefficients are reduced to 1, leaving a single non-dimensional parameter *β*:

(25) $$\frac{dX}{d\tau }=F^{b}+F^{g}+\beta F^{c},$$

where *β* = *ϵ/ζv*_0_ is the ratio between the gliding and cohesion forces and is subsequently referred to as the cohesion strength. (In the subsequent sections, simulations for *β* = 0 use a modified interaction force, such that there is no cohesion, but the hard core repulsion is maintained, *i.e.*, $F_{0}^{c}$ = 0 for *h* *≥* 0, otherwise $F_{0}^{c}$ = *F^c^*with *β* = 0.125).

### 2.8. Parameters

After making the equations of motion independent of scale, we are left with a single non-dimensional parameter, *β*, in the equations. However the reversal frequency *ω/T* is another important parameter that is “hidden” in the stochastic reversals process. The system also has non-dimensional parameters that characterize its configuration and, subsequently, the dynamics: the trichome density (*ρ*), measured as the volume fraction of the domain occupied by the trichomes, the diameter (Θ*/L*) and length of the trichomes (Λ*/L*), the domain dimensions (*W/L*, *H/L*, *D/L*) and the number of segments each trichome is composed of (*N* ).

The parameter values used in our simulations are based on Shepard *et al.* [7] experiments. The *Pseudanabaena* strain used in their study was on average 1.5 µm in diameter and up to “hundreds of micrometers long”. We take Λ = 750 µm, which seems fairly common for filamentous cyanobacteria [41,42], although reports rarely present a quantitative distribution of trichome lengths. The gliding speed was observed to be up to 1.3 µm/s for the *Pseudanabaena* trichomes, and we take this value for *v*_0_.

For the drag coefficient, we follow [36,43,44] and use the analytical expression for the drag coefficient of a thin cylinder divided by the number of vertices per trichome:

(26) $$\zeta =\frac{1}{N+1}\frac{4\pi \mu \Lambda }{\ln(2\Lambda /\Theta )+c},$$

where *c* is of order unity and depends on the shape of the filament and *µ* is the viscosity of the fluid. We assume that the drag coefficient is constant, ignoring the boundary effects of a trichome gliding very close to a wall, as well as hydrodynamical forces between trichomes. We assume *µ* = 1 Pa s [38] and *c* = 1*/*2 [43].

The bending modulus of the trichomes was first estimated using the data presented in Boal and Ng’s [45] study comparing ancient filamentous microfossils to extant cyanobacterial species. Although the authors actually measure the persistence length *ξ* of the trichomes, *ξ* should be directly proportional to the bending modulus *α* = *k_B_Tξ*, if the flexure is due to thermal fluctuations alone. Therefore, with a persistence length of 480 *±* 50 µm, the bending modulus of the *Pseudanabaena* strain used in the study would be approximately 1.94 *×* 10*^−^*^24^ Nm^2^. In practice, we found this value to be too low, as the virtual trichomes would appear completely flaccid and collapse into heaps upon colliding with other trichomes during simulations. Indeed, the implied bending modulus is an order of magnitude smaller than that of *M. xanthus* [38], the diameter of which is less than a fourth of *Pseudanabaena*’s and which also has a “patchy” peptidoglycan layer [46], making it relatively flexible.

How low is this value really? We can calculate a rough estimate of the Young’s modulus of the cell wall as a measure of the material stiffness of the trichomes independent of their diameter. The bending modulus is given by *α* = *Y I* where *Y* is the Young’s modulus and *I* is the second moment of inertia of the cross-section. Assuming that the bulk of the bending resistance lies in the cell wall, such that *I* is equivalent to that of a hollow cylinder, we have *Y* = 4*α/π*(*r*^4^*−* (*r* *−* *t*)^4^) where *r* is the radius of the trichome and *t* is the thickness of the cell wall. Assuming that the cell wall thickness of *M. xanthus* is 2 nm (the lower bound for Gram-negative bacteria [47]) and its diameter is 0.5 µm, a rough estimate of the Young’s modulus of its cell wall is *≈*3.1 *×* 10^5^ Pa. This is two orders of magnitude lower than the benchmark value of 1 *×* 10^7^ Pa for the peptidoglycan layer of various bacteria ([48,49] and the references therein); however, we have already mentioned how Myxobacteria are typically more flexible than other bacteria. Performing the same calculation for *Pseudanabaena*, assuming that the cell wall is 10 nm thick (the lower bound for cyanobacteria, [47]) and a diameter of 2.1 µm, this yields a Young’s modulus of just 54 Pa.

Although the deep constrictions between *Pseudanabaena*’s cells confer it greater flexibility, our initial estimate of the bending modulus seems to be unreasonably low. One possibility is that the bending observed in the trichomes in Boal and Ng’s study was not the sole consequence of random thermal fluctuations, as was assumed, and was exacerbated by cell motility and collisions with neighboring trichomes, thereby significantly underestimating the bending modulus via the relation *α* = *ξk_B_T*. Furthermore, the strains used in the study seem to have been exceptionally fragile, as two of the three strains cultured had to be discarded because of extremely short trichome lengths, and the remaining strain produced very short trichomes of around 40 µm.

We resort to making an educated guess as to the true bending modulus of *Pseudanabaena*. Assuming that the trichomes are on the flexible side of the spectrum, we take 3.1 *×* 10^5^ Pa as a lower bound on the Young’s modulus of the cell wall. Then, with a trichome diameter of 1.5 µm and taking 10 nm as the lower bound of cyanobacteria cell wall thickness, we arrive at *α* = 2 *×* 10*^−^*^21^ Nm^2^ for the bending modulus in our model. This value corresponds to a persistence length of 0.49 m, which implies that the trichomes would be practically unaffected by thermal fluctuations.

The parameters of the model, as well as the values used, are summarized in Table 1.

**Table 1:** Model parameters. The parameters for all of the simulations are set to the values in the table unless otherwise specified in the text. (♭) Extrapolated from various sources; see the text for details.

Type	Symbol	Description	Value	Refs.
Numerical Integration		Absolute accuracy	0.1 µm	
Domain	*W*	Width	3,500 µm	
	*H*	Height	7.5 µm	
	*D*	Depth	3,500 µm	
	*µ*	Viscosity	1 Pa s	[38]
	*ρ*	Trichome density	2.5% vol	
Trichomes	Θ	Diameter	1.5 µm	[7]
	Λ	Length	750 µm	[7]
	*l*	Segment length	*∼*6 µm	
	*α*	Bending modulus	2 *×* 10*^−^*^12^erg*·*cm^−1^	♭
	*v*	Gliding speed	1.3 µm/s	[7]
	*ω*	Reversal frequency	1/300 s ^−1^	[29,50]

### 2.9. Algorithms and Statistics

All simulations were performed using custom-made C++ programs and executed on graphics processing units using the nVidia CUDA SDK version 4.0 provided by the nVidia Corporation (Santa Clara, CA, USA). The *thrust* template library, as well as the CURAND library for random number generation were used. Simulations were executed on nVidia Tesla processing cards using single precision floating point arithmetic.

We experimented with the first order Euler method and the fifth-order Cash–Karp numerical methods, both with adaptive step-size control [51]. Since we are not interested in the exact movement of individual trichomes, but rather the ensemble properties of the system, we decided to use the low order Euler method with a target absolute accuracy of 0.1 µm. We found that the simpler Euler method provided better performance when low accuracy is required.

To enforce the inextensibility of the trichomes, we used the LINCS algorithm [39]. The algorithm was applied after each unconstrained Euler step of the numerical method.

Contact interactions were handled by generating an interaction list consisting of pairs of edges within 2Θ distance of each other (our GPU implementation of this routine was inspired by the “particles” application by Simon Green, which is bundled with the CUDA SDK examples). The lists were reused until a least one vertex had moved Θ distance from its position when the previous list was generated. This effectively means that the Lennard–Jones function was truncated at *h* = 2Θ, at which the force intensity is less than *<*1% of the peak. However, a significant consequence of this computational optimization is that if two edges are outside of each other’s attractive range and are pushed into range through other forces, it is possible that the two edges will not feel an attractive force until the neighbor lists are updated. However, once the update occurs, the edges will always be subject to the attractive force until being separated by other forces. We find that these errors are infrequent and acceptable as a trade off for increased computational performance.

Data analysis was performed using Mathematica 8. For each set of parameter values tested, five simulations with different random initial conditions were performed. The statistics shown in the Results section are the mean of the five simulations, and the error is the standard deviation of the means for each system separately.

## 3. Results

### 3.1. Varying Cohesion Strength

**Figure 3:**
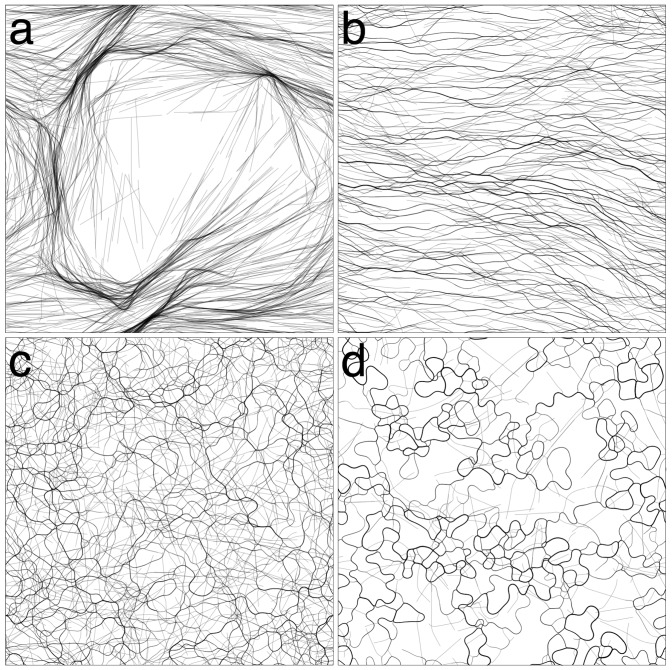
Varying *β* (cohesion/gliding ratio). (**a**) *β* = 0, the trichomes organize into broad streams where they are locally aligned. The streams narrow in places forming dense clusters of trichomes. Streams bend sharply in some places. The streams appear stable at *t* = 12 h; (**b**) *β* = 0.25, the trichomes organize into thin parallel bands that are roughly globally aligned. The distribution of the trichomes appears stable at *t* = 12 h; (**c**) *β* = 1, the trichomes form a chaotic network of thin bands. No discernible macroscopic pattern is observed; (**d**) *β* = 5, virtually all trichomes are banded. The bands are chaotically dynamic, constantly splitting and merging with neighboring bands.

In our initial round of simulations, we tested the effect of cohesion strength on pattern formation. We tested values *β* *∈* {0*,* 0.25*,* 0.5*,* 1*,* 2.5*,* 5}. We only tested up to *β* = 5, since for higher values, the trichomes would quickly form tight clumps and become immotile. For the values tested, three distinct patterns emerged from the simulations, as exemplified in Figure 3. We first describe the patterns qualitatively and then apply various measures to justify our observations.

**Figure 4:**
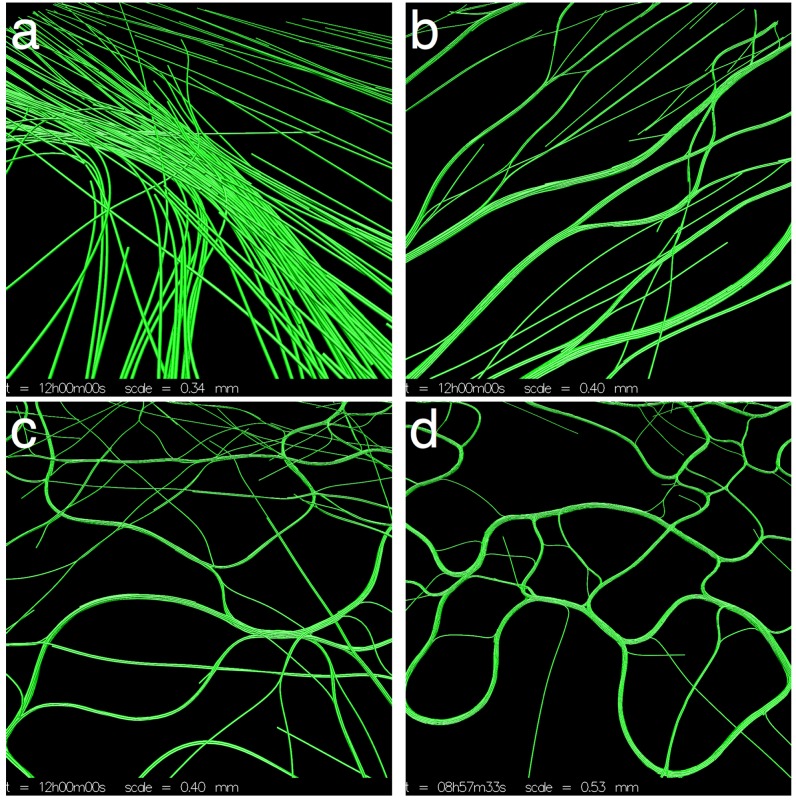
Details of the simulations from Figure 3. (**a**) *β* = 0, the broad streams narrow in some places, forming dense spots; (**b**) *β* = 0.25, the thin parallel bands often branch and merge with others; (**c**) *β* = 1, bands become more sinuous and misaligned; (**d**) *β* = 5, irregular loops dynamically appear and disappear.

For *β* = 0 (Figure 3a, Movie 1 in Supplementary), the trichomes self-organized into broad, locally-aligned streams within 12 h. The topology of the streams appeared to be stable, even though trichomes are constantly passing in and out of them. The pattern is remarkable in that it is static in terms of its macroscopic structure and, yet, is composed of constantly moving elements with no underlying structures or cues to enforce the pattern. At a few points, the streams narrow and form dense constrictions (Figure 4a), which seem to grow increasingly dense over time. Furthermore, the streams bend sharply at some places, forming distinct angles.

For *β* = 0.25 (Figure 3b, Movie 2 in Supplementary), a new pattern of thin elongated bands forms. The bands are all roughly parallel to each other, and so, the system appears to be globally (nematically) aligned. The thickness of the bands varies, and the bands often branch and merge with other bands (Figure 4b). The bands do seem to converge somewhat to form locally-dense constrictions as before, but not to the same extent as with *β* = 0. After 12 h, the patterns appear to be stable, and no rapid changes in the orientation or distribution of the trichomes is discerned.

For *β* *∈* {0.5*,* 1.0}(Figure 3c, Movie 3 in Supplementary), the system becomes seemingly chaotic. Thin bands still form; however, they are now contorted and less aligned with neighboring bands. The bands branch frequently and merge with neighboring bands, forming a complicated network with no obvious regular structure (Figure 4c). Furthermore, the network is not stable in time and is constantly changing topology as bands split or merge with neighboring bands. No stable macroscopic streams appear to form, as in the previous two cases.

For *β* *∈* {2.5, 5.0} (Figure 3d, Movie 4 in Supplementary), cohesion is strong enough that virtually all of the trichomes are located in a band. The bands are very contorted, yet form a coherent “wavy” pattern. The pattern is not static. The bands constantly split and merge with neighboring bands, changing the topology of the pattern. The bands tend to move perpendicularly to their long axis, increasing their curvature as they do. A notable feature of the system is the appearance of irregularly-shaped loops (Figure 4d). The loops tend to expand in size until they spontaneously dissolve, merge with a neighboring structure or become “pinched”, forming two loops.

To quantify the features of the system, we used various different measures: global alignment, local alignment, local cluster size, trichome tangent correlation length, small sector alignment autocorrelation time and the small sector density frequency distribution. Global alignment (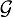) was quantified by calculating the length of the mean direction vector:

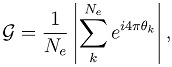
(27)
where *θ_k_* is the angle of the *k*-th edge in the model projected onto the *xy* plane. Local alignment (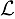) for each edge was calculated by taking the norm of the resultant of all the relative orientations of the edges within a Θ radius:

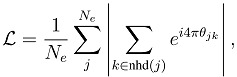
(28)
where *θ_jk_* is the angle between edges *j* and *k* (without projecting onto the *xy* plane). The local cluster size (*C*) was calculated for each edge by counting the number of trichomes within a 2Θ radius. The mean tangent correlation length (*ξ*) was calculated by first calculating the tangent autocorrelation function of each trichome

(29) $${\text{acf}}_{f}(k)=\frac{1}{N-k}\sum_{i=1}^{N-k}e_{t,i}\cdot e_{t,i+k},$$

where *N* is the number of edges per trichome and **e***_f__,i_* is the direction of edge *i* of trichome *f* . The mean tangent correlation length was then calculated by fitting the mean autocorrelation function acf(*k*) to the exponential function *e^−^^ξ k l^*, where *l* is the edge length.

**Figure 5:**
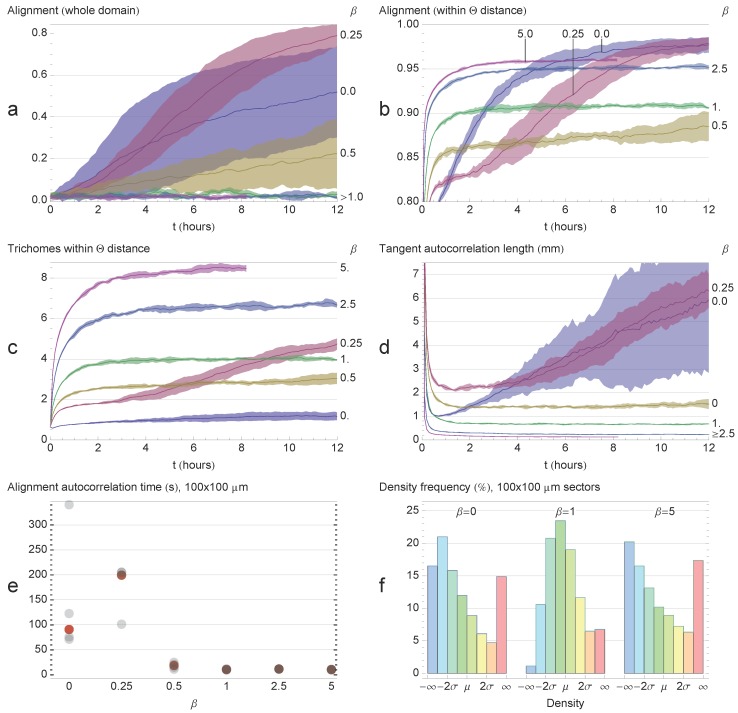
Quantifying system observables. Shaded areas correspond to the standard deviation of the means of five simulations. All simulations ran for 12 h, except for *β* = 5, which ran for 8 h. (**a**) Global alignment. Global alignment decreases with *β*, with the exception of *β* = 0.25, which showed the highest degree of alignment; (**b**) Local alignment. Weak cohesion leads to a high degree of local alignment as the trichomes align in streams and become straighter. Medium values disorganize the system, and high values again lead to high local alignment as the trichomes aggregate into bands; (**c**) Local density increases with cohesion. *β* = 0.25 showing exceptional local density; (**d**) Tangent correlation length decreases with *β*. For low *β*, trichomes become straighter with time; (**e**) Alignment autocorrelation time in 100 × 100 μm sectors over two hours for each simulation. The red dot indicates the median value. Systems with little cohesion show much higher alignment correlation times than systems with significant cohesion; (**f**) Sector density distribution. The initial uniform distribution is Gaussian with mean *μ* and standard deviation *σ*. Self-organization for *β* *∈* {0*,*5}; is indicated by a skew in the density distribution as trichomes aggregate into patterns and depart from the uniform distribution. On the other hand, for *β* = 1, the system has a roughly normal distribution.

The local alignment autocorrelation time (*T*) was calculated by binning the edges into a regular two-dimensional grid composed of 100 ×100 µm sectors. For each sector, the autocorrelation function of the nematic alignment within the sector was calculated over the last 2 h of each simulation with ∆*t* = 288 s. The autocorrelation functions were then averaged over the whole domain, and the autocorrelation time was then calculated in the same way as the tangent autocorrelation function. We also calculated the density distribution of the sectors and compared it with the initial *t* = 0 distribution. The measures are plotted in Figure 5.

Of all the *β* values tested, *β* = 0.25 showed the highest global alignment (Figure 5a), consistent with the observations in Figure 3. The *β* = 0 simulations showed a high degree of alignment variability. Of the five simulations performed, one achieved a high degree of alignment (*≃*0.8), but the remaining four simulations only reached *∼*0.4. For *β* = 0.5, global alignment was very weak, and for *β* *∈* {1*,* 2.5*,* 5}, the alignment of the edges was essentially random. For *β* *∈* {0*,* 0.25*,* 0.5}, global alignment was still increasing after 12 h, and so, it is possible that the system could achieve a high degree of alignment over an extended period of time.

As expected, the edges displayed a higher degree of local alignment than global alignment for all of the *β* tested (Figure 5a). The highest degree of local alignment was observed for *β* *∈* {0*,*0.25}. Local alignment dropped for *β* = 0.5, which is consistent with the observation that the system appears very chaotic for this value. Local alignment increases with *β* for *β* *∈* {0.5*,*1*,* 2.5*,* 5} as more and more trichomes aggregate into bands (Figure 3d).

Local density (Figure 5c) increased proportionally with the relative cohesion strength as the trichomes aggregate into thicker bands. However, *β* = 0.25 again showed exceptional behavior, with local density exceeding what would be expected from the general trend. The mean tangent correlation length (Figure 5d) decreased with increasing *β*, consistent with what was observed in Figure 3, where increasing *β* resulted in wavier bands. However, for *β* *∈* {0*,* 0.25}, the mean tangent correlation length was increasing in time, indicating that the trichomes were becoming straighter.

The small sector alignment autocorrelation time is shown in Figure 5e for each simulation. Higher values indicate the longer persistence of the average direction in the sectors, which, in turn, indicates the stability of the stream pattern. The results show that systems with low cohesion have a much higher alignment autocorrelation, indicating the stability of these patterns relative to higher values of *β*. The sector density distribution is shown in Figure 5f. At *t* = 0, when the trichomes are scattered randomly, the density distribution is Gaussian with mean *µ* and standard deviation *σ*. Self-organization for *β* *∈* {0*,*5} is indicated by a skew in the density distribution as trichomes aggregate into streams/bands and depart from the Gaussian distribution. On the other hand, for *β* = 1, the system is less organized and the density distribution is closer to the initial Gaussian.

### 3.2. Domain Size, Density and Reversal Frequency

**Figure 6:**
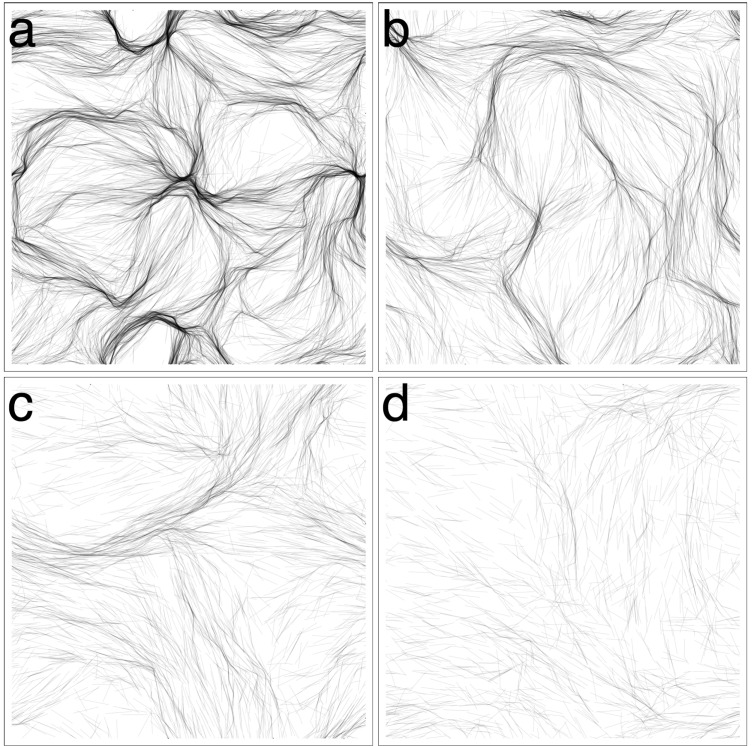
Simulations with no cohesion, a larger domain and variable density. (**a**) *ρ* = 2.5%, trichomes form a network of streams. Streams are broad and ill-defined at some places and narrow and focused at others; (**b**) *ρ* = 1.25%, streams still form, but are less defined; (**c**) *ρ* = 0.625%, the scale of the streams increases; (**d**) *ρ* = 0.3125%, very broad and diffuse streams form.

In this section, we focus on the patterns obtained for *β* = 0 (Figure 3a), which share qualitative similarities with the reticulate mats of [7]. The virtual trichomes form a network of steady streams within the same time frame as the experiments. The size of the domain, however, is not sufficient to get a sense of the broader pattern. Therefore, we performed a new set of simulations with *β* = 0 and a larger 1 *×* 1 cm domain. In addition, we decided to follow-up on the observation that sufficient cell density was required to induce the reticulate pattern in the experiments. We performed simulations for four different densities *ρ* *∈* {0.3125*,*0.625*,* 1.25*,* 2.5}. The results are shown in Figure 6.

For *ρ* = 2.5% (Figure 6a), the same pattern as Figure 3a emerges, but on a larger scale, allowing one to get a better sense of the topology of the interconnected streams. For all values of trichome density tested, the trichomes would form locally-aligned streams. Decreasing trichome density had the effect of broadening the streams and making them less well defined, but streams are nevertheless clearly identifiable (Figure 6b–d). In terms of the measures of the previous sections, the system shows similar behavior as the previous simulations (data not shown). No simulation showed a high degree of global alignment, although the trichomes were locally aligned to a high degree. Local trichome density was directly proportional to the global density, and the tangent correlation length was increasing in time for *ρ* *∈* {0.3125*,*0.625*,* 1.25}, but seemed to stabilize for *ρ* = 2.5% at *≃*4 mm, unlike the previous simulations.

**Figure 7:**
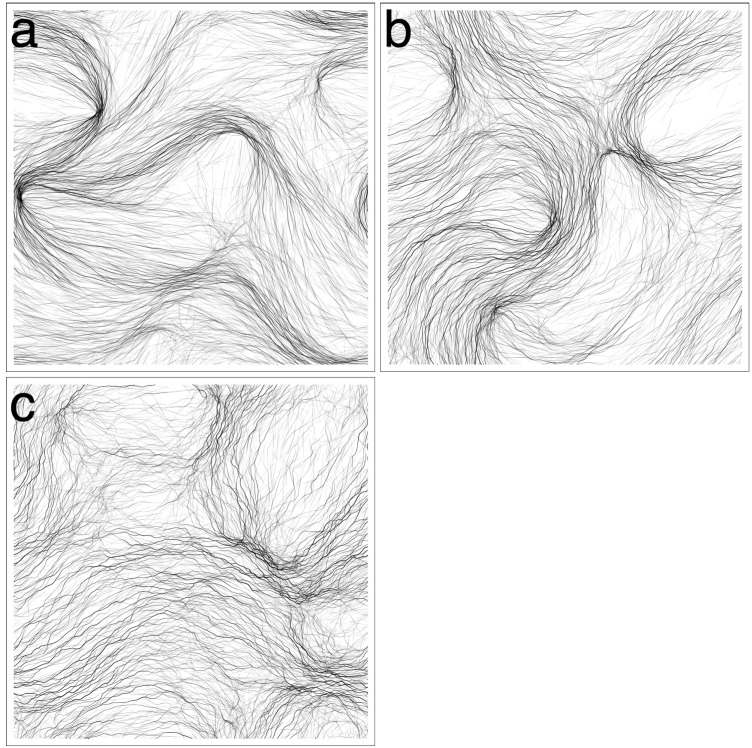
Varying the reversal frequency. The 1 × 1 cm domain. Streaming becomes more indistinct as the reversal frequency is increased. (**a**) *ω* = 0.01 s^-^^1^; (**b**) *ω* = 0.03 s^-^^1^; (**c**) *ω* = 0.09 s^-^^1^.

We also experimented with decreasing the reversal frequency from its typical value (0.003 s*−*1). The results are shown in Figure 7. Although streams are still apparent even for a relatively high reversal frequency (Figure 7b), the pattern is clearly reinforced by more persistent movement.

### 3.3. Revisiting Low Cohesion

**Figure 8:**
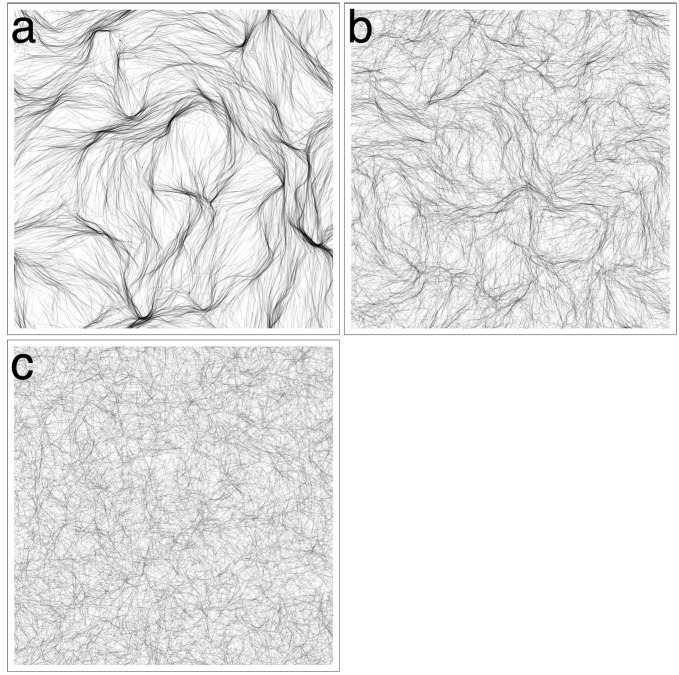
Revisiting low-cohesion in a larger 1 × 1 cm domain. (**a**) *β* = 0.125, streaming occurs although the streams appear more diffuse; (**b**) *β* = 0.25, the global alignment seen in Figure 3b is an artifact of the small domain size. The larger domain used here shows that a streaming pattern still occurs, except streams appear broader and smoother; (**c**) *β* = 0.5, the streaming pattern begins to fade as the system loses its large-scale organization.

An interesting result of the first section was the exceptional behavior seen for *β* = 0.25. The virtual trichomes self-organized into thin, globally aligned bands. On the contrary, for *β* *∈* {0.5, 1}, the system appeared highly disorganized. We performed new simulations with *β* *∈* {0.125, 0.25, 0.5} and a larger 1 × 1 cm domain to see whether we might observe different patterns at a larger scale. The results are shown in Figure 8.

For *β* = 0.125 (Figure 8a), a stable pattern of multiple streams forms; however, the streams are visibly broader and less numerous than in the cohesionless case (Figure 6a). For *β* = 0.25 (Figure 8b), the streams are even broader and have a more homogenous appearance, since there are less constrictions and the streams describe more gentle curves. Clearly, the global alignment in Figure 3b is an artifact of the small domain size. Increasing *β* to 0.5, the stream pattern begins to dissipate (Figure 8c) as the virtual trichomes become more homogeneously distributed in the domain. The simulation results of the previous section for *β >* 0.5 are unlikely to be affected by the domain size, however, since the features in those simulations are on a much smaller scale than the domain size and are also more chaotic, as seen in the correlation length and the global alignment (Figure 5a,d).

## 4. Discussion

We have performed simulations of gliding filamentous cyanobacteria, which typically have a very high length-to-width ratio and are highly motile. We used a cell-based model in which each trichome is explicitly represented as a thin elastic rod. The virtual trichomes glide along their long axis at a fixed speed and periodically reverse direction. We also included a cohesion force, whose strength relative to the gliding force is given by the parameter *β*. This was motivated by the fact that the trichomes exude copious amounts of EPS, which may act as a binding substance.

Many of the model parameters, such as trichome length, diameter, gliding speed, reversal frequency *etc*. are easily measured and are well known. However, the bending modulus is more difficult to measure directly. We attempted to use the bending modulus implied from the relation *α* = *k_B_Tξ* and Boal and Ng’s [45] measurements of the trichome persistence length, but the resulting value seemed too low compared to measurements of other bacteria, and in practice, the virtual trichomes appeared flaccid during simulations. It is possible that the flexure seen in Boal and Ng’s trichomes was due more to the motility of the trichomes than random thermal fluctuations, in which case the above relation would no longer be valid, and a more complex model would be required to associate the observed geometry of the trichomes to their bending modulus. For example, Wolgemuth [38] used an elastic model to estimate the bending modulus of *M. xanthus* by fitting the model to the flailing motions of a Myxobacterium stuck at one end.

In the results, we found that for systems in which gliding overwhelms cohesion, *i.e.*, *β* ≲ 0.5, the virtual trichomes self-organize into a stable pattern of connected streams. The topology of the streams was remarkably stable, and the cohesionless systems (*β* = 0) displayed the sharpest stream patterns. Increasing *β* caused the streams to become broader and smoother. For *β* *∼* 1, in which the cohesion and gliding forces are roughly comparable, the system becomes a chaotic web of trichomes, and no evident pattern emerges. However, by increasing cohesion such that it overwhelms gliding, yet not so strong as to reduce the system to dense clumps (2.5 ≲ *β* ≲ 10), a fine mesh emerges, composed of thin bands of filaments. Unlike in the weak-cohesion systems, the topology of the mesh is dynamic.

The main objective of the model was to capture the behavior reported by Shepard and Sumner [7], who studied the formation of reticulate patterns in cultures of the filamentous cyanobacterium, *Pseudanabaena*, and our simulations bear similarity to their experimental results. As in Shepard and Sumner’s study, virtual trichomes were observed to align upon colliding and formed parallel clumps. In the experiments, a stable reticulate pattern would emerge within hours from an initially homogenous trichome mass inoculated on a submerged substratum. The patterns consisted of a network of ridges formed by densely-packed filaments, each ridge being around 2–5 mm in length and 0.5–2 mm in height. Similarly, in the simulations, we obtain a stable pattern of high density streams that surround low density areas, and the length and time scales of the patterns of both the simulations and experiments are similar. We also observe sharp angles in the system, which may be related to the polygonal forms traced by the ridges in the experiments. Our results indicate that a minimal system of self-propelled, very long trichomes is sufficient to produce a stable pattern consisting of multiple streams in which the trichomes are nematically aligned.

Despite the similarities described above, in the simulations, the streams are less well defined than the ridges seen in Shepard and Sumner’s experiments, and the reticulate formed is irregular compared to the neat polygonal pattern seen in their results (Figure 1). These differences may be due to the fact that we use a shallow domain (7.5 µm) to reduce the simulation run time, which may cause the pattern to be “squashed” as the domain ceiling prevents ridges from growing vertically. Another possibility is that the trichome density we used was too low. Shepard and Sumner [7] found that a minimum trichome density was required for ridges to form. In our simulations, we also found that increasing trichome density reinforces the stream pattern (Figure 6), and it is reasonable to assume that increasing density further would help to consolidate the patterns. For the same reason, including trichome growth and fragmentation in the model might also lead to more robust patterns.

Another aspect to consider is that we have assumed that the virtual trichomes may glide freely in the domain. In reality, gliding only occurs when trichomes are in contact with a solid surface, such as the substratum or another trichome, or embedded in a gel, such as agar. It is not clear whether the EPS produced by the trichomes provides sufficient stiffness or is produced in sufficient volume to allow the trichome to glide freely. Restricting the virtual trichomes such that they may only glide when in contact with the substratum or another trichome may promote further aggregation of the trichomes from streams into ridges, as a trichome would be less likely to successfully break away from a stream, since it would lose much of its propulsive force as it lost contact with neighboring trichomes and/or the substratum.

Finally, it is also possible that our simple model is missing some essential characteristic of *Pseudanabaena*’s behavior. Shepard and Sumner [7] observed that the trichomes were capable of “bending laterally”, but it is not mentioned whether lateral bending has any effect on reticulate formation. *Oscillatoria terebriformis* trichomes exhibit both gliding and flexural movements, and when the trichomes glide against each other, they coil into rope-like structures [52,53]. A similar process may occur in the case of *Pseudanabaena*.

## 5. Conclusions

We have used a cell-based model to study the formation of reticulate patterns in cultures of *Pseudanabaena*. We have found that a minimal system of very long flexible trichomes capable of gliding motility is sufficient to produce stable patterns consisting of a coarse network of streams; however additional features of *Pseudanabaena*’s behavior may be needed to fully explain the emergence of a polygonal reticulum in cultures. An ultrastructural characterization of the reticulum and a detailed and quantitative description of *Pseudanabaena* behavior would certainly add to our understanding of the pattern formation. On the other hand, larger simulations with a higher trichome density and a more realistic domain height may be sufficient to completely reproduce the reticulum.

In future work, we would like to do a quantitative comparison with experimental data and attempt to fit the model parameters to the data. In addition, it would be interesting to test whether this same minimal system together with photomovement is sufficient to produce the cone-shaped structures documented by Walter *et al.* [5] and Petroff *et al.* [6], amongst others. If successful, we could then link macroscopic features of similar stromatolites back to characteristics of the trichomes, as well as improve our understanding of the role of the various processes in building these structures.

## Supplementary Materials

Supplementary materials can be accessed at: http://www.mdpi.com/2075-1729/4/3/433/s1.

## Supplementary Files

Supplementary File 2
